# Evaluation of lung oxidative stress and inflammatory state using exhaled breath condensate analysis in early-life arsenic exposure

**DOI:** 10.1088/1752-7163/ae0510

**Published:** 2025-10-21

**Authors:** Jim K Mansoor, Eva Borras, Emily M Wong, Liliana P Rodriguez, Amanda Silveira, Catterina Ferreccio, Cristina E Davis, Craig Steinmaus, Edward S Schelegle

**Affiliations:** 1 Department of Physical Therapy, School of Health Sciences, University of the Pacific, Stockton, CA, United States of America; 2 Department of Mechanical and Aerospace Engineering, University of California, Davis, Davis, CA, United States of America; 3 Department of Anatomy, Physiology, and Cell Biology, School of Veterinary Medicine, University of California, Davis, Davis, CA, United States of America; 4 Advanced Center for Chronic Diseases, Escuela de Medicina, Pontificia Universidad Católica de Chile, Santiago, Chile; 5 Berkeley Environmental Health Sciences, University of California, Berkeley, CA, United States of America; 6 Arsenic Health Effects Research Group, School of Public Health, University of California, Berkeley, CA, United States of America

**Keywords:** exhaled breath, arsenic, early life exposure, lung function, metabolomics

## Abstract

Millions of people worldwide are exposed to environmental arsenic in drinking water, resulting in both malignant and nonmalignant diseases. Interestingly, early life exposure by itself is sufficient to produce higher incidences of these diseases later in life. Based on the delayed onset of disease, we hypothesized that early life arsenic exposure would also induce long-term alterations in the metabolic profile. The objective of this study was to examine metabolomic biomarkers in exhaled breath condensate (EBC) of individuals exposed to arsenic in drinking water early in life, but not later. One hundred and fifty subjects (75 males and 75 females) were initially recruited from Antofagasta, Chile, some of whom were exposed to high water arsenic levels (⩾870 *µ*g l^−1^; HighAE group), and others, low water arsenic levels (⩽110 *µ*g l^−1^; LowAE group) early in life (1958–1970). EBC samples were collected for targeted and untargeted metabolomic biomarker analysis. The results showed significantly shorter individuals and reduced pulmonary functions (forced vital capacity, FVC and forced expiratory volume in 1 s, FEV_1_) in both males and females in the high-arsenic groups. Males exposed to high arsenic levels also had reduced red blood cell concentrations, as well as higher concentrations of the oxidative stress metabolites 8-OH-2dG and 8-iso-PGF2*α*. Females in the high-arsenic group showed reductions in 8-OH-2dG. Untargeted analysis revealed metabolomic markers that differentiated the HighAE group from the LowAE group, with a subgroup of markers whose concentrations were proportional to the level of arsenic exposure. Targeted and untargeted analyses of EBC using liquid chromatography–mass spectrometry indicated that adults exposed to high arsenic levels in drinking water in utero and during early childhood retained a modified metabolic profile 47 years after the end of exposure.

## 1. Introduction

Millions of people worldwide are exposed to arsenic in food and drinking water [1]. In the United States, an estimated 12% of all public water systems have arsenic concentrations near the current standard (10 *µ*g l^−1^) [2]. Millions of people are exposed to even higher concentrations in unregulated private wells [3, 4]. Ingested arsenic is an established cause of malignant and non-malignant diseases of the lungs and urinary tract, with the developing lung being particularly susceptible. Investigations in Chile involving hundreds of thousands of individuals exposed to a well-documented period of high concentration of arsenic in drinking water, while *in utero* or as young children, but not later in life, have offered an opportunity to investigate the long-term impacts of early life exposure to arsenic on health [5–8]. Some of the pathologies seen due to this exposure include 5-fold increases in lung cancer [7], 3-to 4-fold increases in bladder cancer [8], 46-fold increases in bronchiectasis [9], and 3-to 6-fold increases in respiratory symptoms and lung function decrements, as observed in heavy smokers [10]. This is the first evidence that early life exposure to a common water contaminant can cause major increases in disease in adults [8, 10].

Although there is considerable evidence that the developing lung is highly susceptible to arsenic [5–7], and that early life arsenic exposure is associated with pathological changes in the adult lung and urinary tract [8, 11], the exact mechanisms of the delayed effects are unknown. Oxidative stress, inflammation, and tissue remodeling have been hypothesized to play key roles in arsenic toxicity. Oxidative stress is the overall production of reactive oxygen species (ROS) upon exposure to arsenic [12]. Acute and ongoing chronic arsenic exposure increases oxidative stress markers and is linked to DNA damage, lipid peroxidation, redox enzyme activity, and decreased antioxidant defense levels [12–15]. Arsenic-induced oxidative stress increases the levels of inflammatory mediators. Arsenic exposure has been associated with the pro-inflammatory markers interleukin (IL) 8 and tumor necrosis factor alpha (TNF*α*) in human placenta and cord blood from women chronically exposed to arsenic in drinking water [16]. A pro-inflammatory profile is also seen in women exposed to chronic low-level arsenic in groundwater, as shown by an upregulation of TNF*α* in sputum, increased plasma pro-inflammatory cytokines such as TNF*α*, IL-6, IL-8, and IL-12, and a reduction in the anti-inflammatory cytokine IL-10 [14]. A pro-inflammatory state leads to increased lung tissue remodeling, with increases in markers of remodeling such as matrix metalloproteinase 9 (MMP-9) and tissue inhibitor of metalloproteinases 1 (TIMP-1). Arsenic is associated with increased MMP-9 levels, MMP-9/TIMP-1 ratios, and/or proteinase/anti-proteinase activity in mouse and human lung cells [17, 18]. These changes have also been observed in serum [19] and sputum [14, 17, 20] samples in humans exposed to arsenic. Many of these markers of oxidative damage can be measured in exhaled breath condensate (EBC) [21–23], a simple and non-invasive technique for obtaining information about biomarkers in the airway lining fluid of the lower respiratory tract and systemic circulation, potentially supplying information regarding lung and systemic metabolism.

Unlike many of the studies cited above examining acute or ongoing chronic exposure to arsenic, we examined an adult population exposed to arsenic in utero and in early childhood 47 years after exposure. We used targeted and untargeted metabolomic markers in individuals exposed to arsenic early in life during a well-documented period of high arsenic exposure in northern Chile and compared them with individuals who had very low or no arsenic exposure. Lung and systemic metabolism are difficult to study noninvasively in humans. In this study, we are the first to use a non-invasive sampling technique for EBC collection in combination with liquid chromatography–mass spectrometry/mass spectrometry (LC–MS/MS) and immunoassay analysis to investigate the effect of early life arsenic exposure on targeted measures of oxidative stress, inflammation, and lung remodeling. We also examined EBC samples for metabolomic patterns of EBC samples in an untargeted analysis using LC–MS. We hypothesized that early life exposure to high arsenic levels in drinking water increases oxidative stress, inflammation, and lung remodeling that persists into adulthood, and that these changes can be detected in EBC. We also hypothesized that early life arsenic exposure would alter metabolic pathways and that this change would be seen in the EBC sample metabolic signature.

## 2. Materials and methods

### 2.1. Study location

This study was conducted in the northern Chilean city of Antofagasta (current population approximately 380 000 [24]). Northern Chile is one of the driest places on Earth, and most people in this region rely on municipal water supplies with measured levels of arsenic dating back to the 1950s [5]. The known sources of municipal water and consistent arsenic measurements over the years allow for the calculation of individuals’ lifetime arsenic exposure by identifying the cities in which they lived. In the 1950s the arsenic concentration in the drinking of the rapidly growing city of Antofagasta, Chile was approximately 90 *µ*g l^−1^, which is below the current World Health Organization guidelines of 110 *µ*g l^−1^ [25]. This limited water supply originated from the Siloli catchment located near the headwaters of the Rio Silala on the border of Chile and Bolivia [26]. In order to meet the increasing water demand of Antofagasta in the 1950s the water supply to Antofagasta was increased in 1958 via a 300 km pipeline that originated at the Toconce catchment at the head waters of the Rio Salado [26]. While markedly increasing the water supply to Antofagasta, the water from the Toconce catchment increased the water concentration of arsenic approximately nine times to 860 *µ*g l^−1^. The arsenic concentration remained elevated at this high level until May of 1970 when a large water treatment plant using iron chloride as a coagulant agent became operational [5, 26]. Since then, arsenic concentrations have been below 110 *µ*g l^−1^, with current levels of around 10 *µ*g l^−1^ [26]. This exposure scenario caused a large group of people who were born in 1958 and after to be exposed to high levels of arsenic in utero and as young children, but not as adults. The water originating from the Siloli and Toconce catchments have a high pH (>8.0) and contain other inorganic solutes other than arsenic including calcium, magnesium, sodium, chloride, sulfate, bicarbonate, silicone oxide, boron, lithium, and strontium, at similar concentrations. Neither source contained other metals or trace elements such as selenium, molybdenum, barium, manganese, lead or mercury that could contribute to the symptoms and health decrements reported in Antofagasta after 1958 [26, 27].

### 2.2. Subjects and study design

A total of 150 participants (75 females and 75 males) aged 47–58 years were recruited through personal communication and fliers posted by employees of the regional hospital of Antofagasta (Hospital Regional de Antofagasta). Recruitment started on 27 April 2017, and continued until 7 July 2017. The study protocol was approved by the institutional review board of the University of California, Berkeley (protocol number 2010–04–1140). The study was approved by the Pontificia Universidad Católica de Chile (protocol number 170 127 001). All participants provided written informed consent before participation.

Subjects were paid for approximately 4 h of their time. Subjects were interviewed by one of two Chilean nurse investigators while filling out a questionnaire, which included information on lifetime residential history, water intake, smoking, diet, occupation, respiratory symptoms, and past diseases. On a subsequent visit to a medical clinic from a regional hospital, examinations, spirometry, and biological sample collection were performed. The height and weight of each subject were measured, and blood and urine samples were collected. The subject then underwent a pulmonary function test. After this test, each subject sat quietly for up to 50 min in an EBC system to collect an EBC sample. This design allowed us to examine individuals who were born in Antofagasta and exposed to early life high arsenic levels and compare them with individuals who were born elsewhere and/or had much lower arsenic exposure early in their lifetimes.

### 2.3. Subject questionnaire

Each participant was administered a structured questionnaire to assess their lifetime residential and occupational history (all jobs or residences occupied ⩾6 months), water source types (municipal tap water, bottled, other), current medications, and medical history. Smoking histories included age at start and cessation, years smoked, and average number of cigarettes smoked per day. Ever-smoking was defined as smoking cigarettes at least once per week for ⩾1 year, or 20 packs lifetime. Secondhand smoke was defined as someone smoking in the same room at home or at work. Indoor air pollution was defined as irritating or visible smoke, vapors, gases, or dust in the same room. The participants were also asked about the types of fuels used at home. Occupational exposure was defined as regular exposure to vapors, dust, gas, or fumes in a job held for ⩾6 months [28]. Standardized questions were adapted to local Spanish from questionnaires used by the Latin American Project for the Investigation of Obstructive Lung Diseases (PLATINO), the third U.S. National Health and Nutrition Examination Survey (NHANES III), and the second European Community Respiratory Health Survey (ECRHS II).

Questions about respiratory symptoms were adapted from the British Medical Research Council [29]. Participants were asked, ‘Do you often cough when you do not have a cold, such as in the mornings in winter?’ Chronic cough was assessed using the follow-up question, ‘Do you cough like this for at least 3 months a year?’ The same questions were asked about phlegm. The participants were also asked whether they had trouble breathing (1) rarely, (2) often, or (3) always. Finally, participants were asked whether they became breathless when (1) hurrying on level ground or walking up a slight hill, (2) walking with other people of the same age on level ground, or (3) they had to stop for breath when walking on level ground at their own pace.

### 2.4. Arsenic exposure assessment

Age-specific estimates of arsenic exposure from municipal drinking water were assessed using birth dates, residential history, and available arsenic water records for Chilean cities and regions [5, 7, 9]. The municipal water database included over 15 000 arsenic measurements from Antofagasta and 11 other cities in northern Chile between 1930 and 1995, when concentrations transitioned from low to high to low. Low arsenic exposure in early life (LowAE) was defined as drinking water containing ⩽110 *µ*g l^−1^ arsenic at birth, ⩽1000 *µ*g l^−1^ years cumulative arsenic from age 0–10 years, and ⩽2000 *µ*g l^−1^ years cumulative arsenic from age 0–20 years. Arsenic levels above these were considered as high arsenic exposure (HighAE). Sensitivity analysis was conducted to evaluate which of these exposure metrics had the strongest association with pulmonary function and targeted EBC measures. Exposure response was assessed by using early life arsenic concentration as a continuous variable and by stratifying subjects into low- and high-exposure categories.

### 2.5. Lung function testing

After height and weight were measured by nurse investigators, lung function was assessed as previously described [10] following the American Thoracic Society/European Respiratory Society guidelines [30] using an EasyOne spirometer (NDD Medical Technologies, Zurich, Switzerland) in diagnostic mode. The same trained nurse investigator used the same spirometer for all subjects. Each subject’s best trial (the largest sum of forced vital capacity (FVC) and forced expiratory volume in 1 s (FEV_1_)) was included in the analyses. Predicted values were calculated using equations obtained from LeVange *et al* [31] for a South American population. Pulmonary function residuals were calculated as *absolute (measured) values—predicted values*.

### 2.6. Sample collection

**Figure 1.**
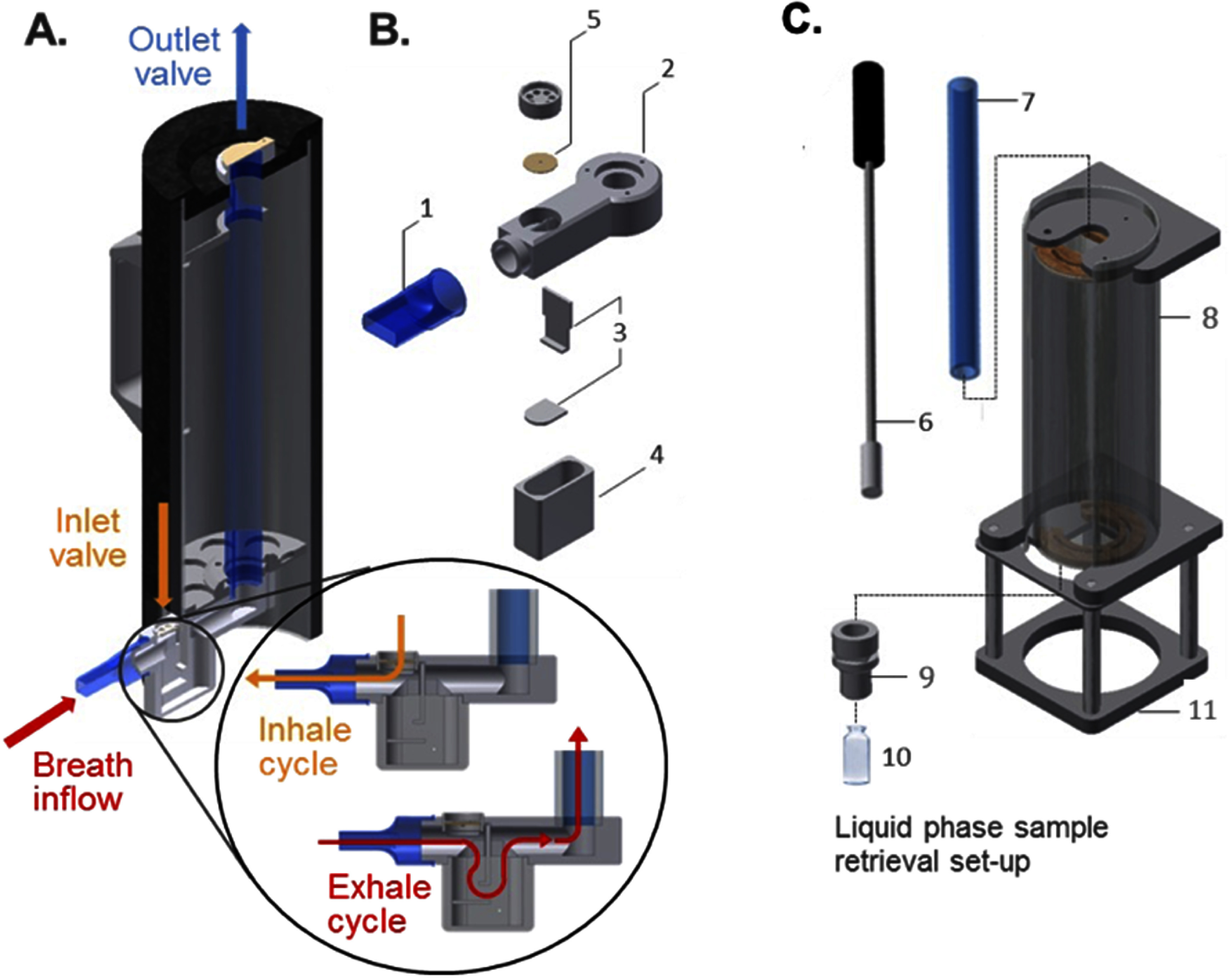
Exhaled breath condensate sample collector unit. A. CAD model of the human breath sampler. Main parts: saliva trap assembly, condenser tube, insulated ice bath housing. Inset circle shows the breath flow diagram for inhale and exhale cycles. B. Detailed view of saliva trap components: 1. mouthpiece, 2. saliva trap housing, 3. trap notch, 4. saliva reservoir, 5. inhale valve. C. Sample retrieval press set-up components: 6. stainless steel sample retrieval plunger with PTFE head, 7. condenser glass tube, 8. vertical retrieval press housing, 9. threaded vial holder, 10. SUPELCO glass vial, 11. vertical stand.

EBCs were collected using a custom device (figure 1) developed previously [32]. Briefly, a condenser glass tube was attached to the valves above and below and placed in the middle of a larger insulated housing so that the glass tube could be surrounded by dry ice. Subjects breathed through a mouthpiece connected to a valve at the bottom of the condenser glass tube, which allowed ambient air to flow into the subject’s lungs, while exhaled breath flowed through a glass tube surrounded by dry ice. In this manner, the exhaled breath rapidly cooled and froze inside the condenser glass tube. The subjects wore a nose clip while breathing quietly on the system twice for 25 min each time, with a 5 min break between the sample collection periods.

The condenser glass tube was carefully removed from the insulated ice bath housing. The sample was allowed to briefly thaw, and a plunger was used to push the sample out of the condenser glass tube into a sample glass vial (borsalite glass vial). The electrical conductivity of the samples were measured to estimate the electrolyte levels. The condenser glass tube was then rinsed with 4 ml of ethanol to obtain the nonwater-soluble fraction of the EBC in a different sample glass vial. All glass vials were weighed before and after the sample was obtained to obtain the sample weight. The sample volume was approximately 4–11 ml for most participants. The samples were then immediately aliquoted into 3–4 different sample tubes: tubes 1 and 2 contained 1 ml of sample mixed with a mixture of 50 ml of internal standards (ISs; see below), while tube 3 contained 1 ml of un-spiked sample. Sample tube 4 contained any leftover EBC sample and tube 5 contained an ethanol wash. Immediately after aliquoting, the samples were placed on dry ice and stored in a −80 °C freezer.

### 2.7. EBC sample analysis

The samples were analyzed for targeted biomarkers of oxidative stress, inflammation, and remodeling using LC–MS/MS or immunoassay. Additionally, using an untargeted approach, metabolic contents were analyzed and assessed as potential biomarkers of arsenic exposure using LC–MS. The ethanol wash samples were analyzed using an untargeted methodology.

### 2.8. Targeted EBC analysis using LC–*MS/MS*

#### 2.8.1. Standards and calibration curve

Compounds related to oxidative stress and inflammation were analyzed using LC–MS/MS (listed in table 1). Compound standards were obtained from Cayman Chemicals (Ann Arbor, MI, USA), except for Dl-o-tyrosine (TCI America, Portland, OR, USA). All solvents and chemicals were HPLC grade or higher (Fisher Scientific, Fair Lawn, NJ, USA).

**Table 1.** Targeted compound standards.

Compound	RT (min)	Precursor ion	Product Ion	CE (V)	ESI	Internal standard
o-tyrosine	0.49	182	136	3	Positive	o-tyrosine-d4
8OH2dG	0.49	282	192	12	Negative	8OH2dG-15N5
RvE1	5.59	349	195	4	Negative	LXA4-d5
11-dh-TXB2	6.49	367	161	12	Negative	11-dh-TXB2-d4
8isoPGF2*α*	6.36	353	193	20	Negative	8isoPGF2*α*-d4
LTE4	7.97	438	333	10	Negative	LTE4-d5
LXA4	7.03	351	115	4	Negative	LXA4-d5
15-HETE	9.20	319	226	2	Negative	15-HETE-d8

Compounds are listed with the corresponding retention time (RT), precursor and product ions, collision energy (CE), ionization mode, and internal standard used for LC–MS/MS analysis.

Calibration curves were calculated using the analyte to IS ratio and a 1/*x* weighting scheme. For each native compound, a suitable IS was selected based on the structural correspondence. Individual stock solutions and mixtures were prepared in a solvent mixture of water: acetonitrile (ACN): isopropanol (IPA) (50:25:25) to obtain final concentrations between 100 and 0.1 ng ml^−1^. Calibration curves, limits of detection (LOD), and quantification were determined for each compound, and each sample was injected in duplicate. The method was validated using quality controls (QCs) prepared with 1 ml of pooled EBC obtained from healthy control subjects and spiked with six different concentrations of standards. Each QC was prepared in each batch following the same procedure used to prepare the samples.

#### 2.8.2. Sample preparation

Frozen samples containing 1 ml of EBC were lyophilized overnight to remove water. The dry extracts were then reconstituted in 60 *µ*l of a solvent mixture of water/ACN/IPA. The glass vials were placed into 50 ml Falcon tubes and spun down at 5000 rpm for 1 min. Samples were then transferred into autoclaved 2 ml tubes with spring inserts and sonicated in an ice water bath for 10 min at 0 °C, removed from the water bath, and centrifuged again at 13 000 rpm for 10 min at 4 °C. Finally, the samples were transferred into microvolume inserts and stored at −80 °C until LC–MS/MS analysis. All samples were prepared in batches of 40–50 samples to assure their stability.

#### 2.8.3. LC–MS/MS analysis

Samples were analyzed using an Agilent Infinity 1290 liquid chromatograph system coupled to an Agilent 6490 Triple Quadrupole mass spectrometer with a Dual Agilent jet stream electrospray ionization source (AJS-ESI) (Agilent Technologies, Santa Clara, CA, USA). Chromatographic separation was performed on an Agilent InfinityLab Poroshell 120 EC-C18 column (50 mm × 3.0 mm 2.7 *µ*m particle size) held at 45° C with an injection volume of 20 *µ*l for each run. The mobile phases consisted of (A) water and (B) acetonitrile/isopropanol (50:50), both in 0.1% acetic acid. A gradient was used to separate compounds at 0.6 ml min^−1^ of 16 min. Analytes were measured using the multiple reaction monitoring mode in a single run, combining positive and negative ionization modes.

Data were initially checked for qualitative purposes using Agilent’s Mass Hunter Qualitative Analysis B.06.00 software. Subsequently, Agilent’s Mass Hunter Quantitative (QqQ) Analysis B.07.00 software was used for the targeted approach using LC–MS/MS analysis of EBC. The compounds were identified, confirmed, and integrated using ion masses, retention times, and MS/MS information. Quantification was performed using standard calibration curves with IS corrections as previously described [33]. Controls were used to validate calibration. The final concentrations were corrected by the initial volume of EBC used before sample preparation, and the results were expressed in pg ml^−1^ of EBC.

### 2.9. Targeted EBC analysis using immunoassays

Sample concentrations of interleukin (IL)-6, IL-8, and tumor necrosis factor (TNF)-*α* were determined using a commercially available Luminex® multiplex bead-based kit (Human Magnetic Luminex Performance Assay Base Kit A; R&D Systems, Minneapolis, MN, USA), in accordance with the kit-specific protocols provided by R&D Systems. The plates were read and analyzed using a Bio-Plex 200 system (Bio-Rad Laboratories, Hercules, CA, USA) and companion software. A five-parameter model was used to calculate the final cytokine concentrations and values (expressed in pg ml^−1^). Additionally, sample concentrations of matrix metalloproteinase-1 (MMP-1) and tissue inhibitor of metalloproteinase-1 (TIMP-1) were assessed in one-third of the samples; however, concentrations were below the level of detection, and thus, the remaining samples were not analyzed.

### 2.10. Untargeted EBC analysis using LC–MS

#### 2.10.1. Chemicals and reagents

Acetonitrile (ACN), water, acetic acid, formic acid, ammonium format, and ammonium acetate were obtained from Fisher Scientific (Fair Lawn, NJ, USA). All solvents and chemicals used were of HPLC grade or higher.

#### 2.10.2. Sample preparation

The remaining EBC sample volumes were used for untargeted analysis. Although most samples had 2 ml of EBC, when the remaining volume was low, we used 1 ml of EBC. In both cases, the volumes were measured from thawed samples and immediately re-frozen at −80 °C. After a minimum of 1 h, frozen samples were lyophilized overnight. The dried extracts were reconstituted in a solvent mixture of water/acetonitrile (90/10). 50 *µ*l were used to reconstitute when the initial EBC volume was 1 ml, and 100 *µ*l was used for higher volumes of 2 ml. Reconstituted extracts were centrifuged and sonicated as described above for targeted analysis. Final volumes were transferred into inserts and stored at −80 °C until LC–MS analysis. Similarly, all samples were prepared in batches of 40–50 samples to assure their stability.

#### 2.10.3. Ethanol-wash fraction preparation

For the ethanol-washed fractions collected after EBC, we used all available volumes (2–4 ml). The vials were completely dried using nitrogen steam. The resulting extract was then reconstituted with 50 *µ*l of water/acetonitrile (90:10), centrifuged, sonicated, and transferred, as described for the EBC fractions. Ethanol wash samples were stored at −80 °C until LC–MS analysis.

#### 2.10.4. Instrumental LC–MS analysis

Untargeted sample analysis was performed using an Agilent 1290 series ultrahigh-performance LC system coupled with an Agilent 6230 time-of-flight mass spectrometer (Agilent Technologies, Santa Clara, CA, USA). To obtain a wide coverage of non-volatile compounds, two complementary analytical columns were used: a reversed phase (RP), for molecules with lower polarity, and hydrophilic interaction chromatography (HILIC), for hydrophilic molecules with higher polarity. Samples reconstituted in 50 *µ*l (EBC and EtOH wash) were only analyzed in RP, and samples brought to 100 *µ*l were run in both HILIC and RP. The LC–MS analytical conditions are described elsewhere [34]. The LC–MS data analysis process involving deconvolution, alignment, and tentative compound identification has also been described in previous studies [35].

Raw peak tables were first merged by ionization mode, obtaining three different datasets based on the sample type (EBC or EtOH) and analysis method (HILIC or RP): EBC/RP samples, EtOH/RP samples, and EBC/HILIC samples. For each dataset, features were filtered by cleaning signals appearing in blank samples with signals higher than 10 (peak sample/blank ratio), by removing missing features in more than 20% of the samples, as well as variables with low variability (RSD < 5% in all samples). To remove systematic bias between measurements, the samples were normalized using the original sample volume per sample. All missing values were replaced by LOD/10 or the minimum positive value divided by 10. Heteroscedasticity was corrected in the final dataset using a log transformation.

### 2.11. Statistical analysis

#### 2.11.1. Number of subjects included in statistical analysis

A total of 150 participants were initially enrolled in the study. Of these 150 subjects, six produced no or insufficient volume of EBC for analysis and were excluded from the analysis. An additional six subjects produced EBC with conductivity values consistent with significant contamination with saliva and were excluded from the analysis. As a result, data from 138 subjects were used for the statistical analysis.

#### 2.11.2. Targeted marker analysis

Continuous anthropometric, pulmonary function, blood parameters, and targeted EBC data were initially analyzed using a multivariate 3-way ANOVA using sex (male vs female), smoking (smoking score, pack-year score, or maximum cigarette score), and arsenic exposure (at birth, cumulative arsenic from age 0–10 years, or cumulative arsenic from age 0–20 years) (SPSS, IBM Inc.). A comparison of these tests indicated that the smoking score (never, former, and current smokers) and cumulative arsenic from age 0–20 years (<2000 mg ml^−1^ years vs ⩾2000 mg ml^−1^ years) best fit the data. Fit and power were further improved by excluding former smokers (25 subjects). This improvement in fit and power was the result of only four female former smokers with high arsenic exposure and only five male former smokers with low arsenic exposure. Based on this initial analysis, further statistical analysis was performed using the remaining data from 113 subjects. If we obtained a significant p-value (⩽0.05) for any of the final 3-way ANOVAs examining the anthropometric, pulmonary function, blood, and targeted EBC parameters, the effect of arsenic exposure within subgroups for sex (males and females) and smoking (never smokers and current smokers) was examined. Categorical data, including respiratory disease scores and occupational exposure to particulates and fumes, were analyzed using Wilks’ *G* test.

#### 2.11.3. Untargeted marker analysis

Data stratified based on sex (male vs female), smoking history (never vs current), and arsenic exposure (low vs high) were analyzed using Excel, MATLAB R2017a, and PLS Toolbox V8.6.2 software for univariate and multivariate analyses. Parametric and non-parametric tests (*t*-test, Wilcoxon rank sum test, or ANOVA/one-way Kruskal–Wallis test) were used to compare means and assess the significance of the fold change (FC) of the resulting features. FC and p-values were combined using volcano plots, allowing an initial variable selection by identifying features with significant differences between the groups studied. P-values lower than 0.05 and FC higher than 2 were used as criteria.

For comparative purposes, two multivariate analysis methods, principal component analysis (PCA) and partial least-squares discriminant analysis (PLS-DA), were applied to the EBC/HILIC (*n* = 73), EBC/RP (*n* = 107) and EtOH/RP (*n* = 106) alone, and with all sample/methods combined (*n* = 66). The number of samples available for analysis for each sample/method combination varied based on the total volume of samples available for analysis obtained from each subject. PCA was performed to visualize the similarities between observations and detect potential outliers in an unsupervised manner. PCA projects the maximum variance of the dataset in a linear additive model. Arsenic a classification method, we used a PLS-DA-supervised approach that uses the correlation between the dataset of features and a matrix of known responses that contains sample information and classes/groups. Partial least-squares DA separates the different groups of samples based on their features. Classification ability is defined by the accuracy or area under the curve (AUC) values, sensitivity (probability of correctly detecting a class), and specificity (probability of correctly rejecting a class). Receiver operating characteristic curves provide AUC values that measure the classification ability at different thresholds, indicating the extent to which a model is able to distinguish classes or groups. Each PLS-DA model ranks features using variable importance in projection (VIP) values. Variable importance in projection values summarizes the impact of each feature in the model, and values higher than one are considered relevant for that classification. The identification of features with a VIPs > 1 provides valuable information on potential markers. Using the peaks identified in the PLS-DA, potential markers of arsenic exposure with no overlap with sex or smoking class were examined using stepwise multivariate linear regression (XLSTAT, Lumivero Inc.), where the continuous value of cumulative arsenic exposure from age 0–20 years was used as the dependent variable.

## 3. Results

### 3.1. Targeted EBC analysis

Table 2 shows subject characteristics broken down by arsenic exposure and sex. Both our LowAE and HighAE groups contained similar numbers of males and females, but our HighAE group had over three times as many subjects in the group. The groups also differed in height and absolute (measured), predicted and residual values of FVC and FEV for 1 s (FEV_1_), with the HighAE group being significantly less than the LowAE group. Table 3 shows the number of subjects who had workplace exposures to particulates and/or chemicals during their lifetimes, with no differences in workplace exposures between the two groups.

**Table 2.** Subject characteristics.

	Average cumulative arsenic exposure: 0–20 years of age						
	0–2000 *μ*g l^−1^ years			>2000 *μ*g l^−1^ years			
	All	Male	Female	All	Male	Female	
	(*n* = 27)	(*n* = 13)	(*n* = 14)	(*n* = 86)	(*n* = 39)	(*n* = 47)	*p* value**
Cumulative arsenic exposure: 0–20 year (*µ*g • years l^−1^)	428 ± 689	269 ± 551	557 ± 788	6327 ± 2763	6354 ± 2793	6304 ± 2768	<0.001
Age (years)	52.2 ± 3.7	52.5 ± 4.2	51.9 ± 3.3	53.7 ± 3.5	53.7 ± 3.8	53.6 ± 3.3	0.065
Height (m)	1.65 ± 0.10	1.73 ± 0.06	1.58 ± 0.04	1.61 ± 0.10	1.69 ± 0.067	1.54 ± 0.07	0.051
Weight (kg)	80.2 ± 14.8	89.2 ± 13.4	71.8 ± 10.6	75.0 ± 13.1	79.8 ± 13.4	70.9 ± 11.5	0.084
BMI (kg m^−2^)	29.3 ± 4.3	29.7 ± 4.0	28.9 ± 4.7	28.9 ± 4.5	28.0 ± 3.8	29.7 ± 4.9	0.685
Smoking							
Current: n (%)	16 (59.3)	9 (69.2)	7 (50.0)	33 (38.4)	18 (46.2)	15 (31.9)	—
Pack-years	3.92 ± 5.88	5.66 ± 6.96	2.31 ± 4.31	4.08 ± 8.71	5.56 ± 9.61	2.85 ± 7.78	0.928
Second hand smoke exposure							
Child: ⩽18 year (years)	4.41 ± 6.63	5.15 ± 6.84	3.71 ± 6.60	3.88 ± 6.56	4.13 ± 6.78	3.68 ± 6.49	0.743
Adult: >18 year (years)	0.00 ± 0.00	0.00 ± 0.00	0.00 ± 0.00	1.78 ± 6.97	0.28 ± 1.76	3.02 ± 9.15	0.104
Respiratory disease (reported)							
Any: n (%)	2 (7.4)	0 (0.0)	2 (14.3)	12 (14.0)	3 (7.7)	9 (19.1)	—
Chronic bronchitis: n (%)	1 (3.7)	0 (0.0)	1 (7.1)	9 (10.5)	2 (5.1)	7 (14.9)	—
Symptoms: cough; wheeze; phlegm, SOB: n (%)	1 (3.7)	1 (7.7)	0 (0.0)	9 (10.5)	2 (5.1)	7 (14.9)	—
Pulmonary function							p value*
FVC (l)	3.90 ± 0.92	4.46 ± 0.91	3.37 ± 0.55	3.44 ± 0.84	4.01 ± 0.67	2.96 ± 0.63	0.008
FVC (% predicted)	100.4 ± 16.7	95.4 ± 14.5	105.1 ± 17.8	94.3 ± 14.9	92.1 ± 13.0	96.2 ± 16.2	0.037
FVC (residual)	−0.009 ± 0.640	−0.188 ± 0.687	0.156 ± 0.567	−0.218 ± 0.551	−0.352 ± 0.596	−0.107 ± 0.490	0.050
FEV_1_ (l s^−1^)	3.06 ± 0.65	3.55 ± 0.50	2.60 ± 0.39	2.65 ± 0.69	3.05 ± 0.69	2.32 ± 0.49	0.004
FEV_1_ (% predicted)	99.2 ± 12.6	96.8 ± 9.4	101.4 ± 15.0	91.9 ± 17.4	88.8 ± 17.3	94.6 ± 17.1	0.024
FEV_1_ (residual)	−0.036 ± 0.366	−0.109 ± 0.336	0.032 ± 0.393	−0.244 ± 0.518	−0.386 ± 0.603	−0.127 ± 0.405	0.028

Values are means ± SD.

Abbreviations: body mass index; SOB: shortness of breath; FVC: forced vital capacity; FEV_1_: forced expiratory volume in 1 s.

Residual = absolute-predicted.

**p value: two-tailed Student’s *t*-test, low arsenic vs high arsenic exposure.

*p value: one-tailed Student’s *t*-test, low arsenic vs high arsenic exposure.

**Table 3.** Workplace exposures to particulates and/or chemicals.

	Average cumulative arsenic exposure: 0–20 years of age				
	0–2000 *μ*g l^−1^ years		>2000 *μ*g l^−1^ years		
	Male	Female	Male	Female	
	(*n* = 13)	(*n* = 14)	(*n* = 39)	(*n* = 47)	*p* value^*^
Mining/smelter (n)	3	1	12	0	0.911
Welding fumes (n)	0	0	2	0	0.294
Solvants (n)	0	0	2	0	0.294
Particulates (n)	3	0	3	0	0.153
Metals (n)	0	0	2	0	0.294
Radon (n)	0	0	0	0	1.000
Pesticides (n)	1	1	1	0	0.111
Other chemicals (n)	6	4	15	15	0.839

^*^ *p* value: Wilks’ G squared test, low arsenic vs high arsenic exposure.

Table 4 shows the results of the 3-way ANOVA examining the main effects of arsenic exposure, sex and smoking history and 1-tailed *T* tests for average cumulative arsenic exposure in the sex (male and females) and smoking (never smokers and current smokers) subgroups. The 3-way ANOVA was used to examine the overall global effect of our independent variables (arsenic exposure [0–20 year], sex, and smoking history) on the dependent variables. The 3-way ANOVA was statistically significant for weight and height, for all pulmonary function measures except FEV1/FVC, for red blood cell and hemoglobin concentrations, hematocrit, and for two measures of oxidative stress, 8-OH-2dG and 8-iso-PGF2*α*.

**Table 4.** Results of 3- way ANOVA (arsenic exposure [0–20 year], sex, smoking history) and average arsenic cumulative exposure (0–20 year) in subgroups.

	3-way ANOVA *p value*	1 tailed *T*-test for average arsenic cumulative exposure (0–20 year) in subgroups											
Variable		Males (*n*)			Females (*n*)			Never smokers (*n*)			Current smokers (*n*)		
		Low arsenic (13)	High arsenic (39)	*p* value	Low arsenic (14)	High arsenic (47)	*p* value	Low arsenic (11)	High arsenic (53)	*p* value	Low arsenic (16)	High arsenic (33)	*p* value
Age (years)	ns	52.5 ± 4.2	53.7 ± 3.8	—	51.9 ± 3.3	53.6 ± 3.3	—	52.1 ± 3.8	53.7 ± 3.2	—	52.3 ± 3.8	53.6 ± 4.03	—
Weight (kg)	<0.001	89.2 ± 13.4	79.8 ± 13.5	0.017	71.8 ± 10.6	70.9 ± 11.5	ns	78.4 ± 10.5	75.2 ± 13.5	ns	81.4 ± 17.3	74.6 ± 12.7	0.063
Height (m)	<0.001	1.73 ± 0.06	1.68 ± 0.07	0.017	1.58 ± 0.04	1.55 ± 0.07	0.059	1.62 ± 0.08	1.60 ± 0.10	ns	1.68 ± 0.10	1.63 ± 0.09	0.045
BMI (kg m^−2^)	ns	29.7 ± 4.0	27.8 ± 3.8	—	28.9 ± 4.7	29.7 ± 4.9	—	30.0 ± 3.6	29.4 ± 4.6	—	28.8 ± 4.8	28.2 ± 4.3	—

Pulmonary function													

FVC (l)	<0.001	4.46 ± 0.91	4.01 ± 0.67	0.039	3.37 ± 0.55	2.96 ± 0.63	0.009	3.68 ± 0.70	3.37 ± 0.84	ns	4.05 ± 1.04	3.56 ± 0.82	0.040
FVC (% predicted)	0.036	95.4 ± 14.5	92.1 ± 13.0	ns	105.1 ± 17.8	96.2 ± 16.2	0.041	102.3 ± 17.8	94.6 ± 15.3	ns	99.2 ± 16.4	93.9 ± 14.4	ns
FVC (residual)	0.011	−0.188 ± 0.687	−0.352 ± 0.596	ns	0.156 ± 0.567	−0.107 ± 0.490	0.047	0.034 ± 0.608	−0.203 ± 0.561	ns	−0.040 ± 0.679	−0.242 ± 0.542	ns
FEV_1_ (l s^−1^)	<0.001	3.55 ± 0.50	3.05 ± 0.69	0.012	2.60 ± 0.39	2.32 ± 0.49	0.016	2.92 ± 0.62	2.62 ± 0.69	ns	3.15 ± 0.68	2.70 ± 0.70	0.019
FEV_1_ (% predicted)	0.029	96.8 ± 9.4	88.8 ± 17.3	0.060	101.4 ± 15.0	94.6 ± 17.1	ns	101.1 ± 10.0	93.0 ± 17.7	ns	97.9 ± 14.3	90.2 ± 16.9	ns
FEV_1_ (residual)	0.006	−0.109 ± 0.336	−0.386 ± 0.603	0.062	0.032 ± 0.393	−0.127 ± 0.405	ns	0.027 ± 0.306	−0.208 ± 0.518	ns	−0.079 ± 0.407	−0.302 ± 0.521	ns
FEV_1_/FVC (%)	ns	80.5 ± 7.1	75.6 ± 9.8	—	77.5 ± 7.9	78.4 ± 6.2	—	79.7 ± 9.2	77.7 ± 6.3	—	78.4 ± 6.5	76.2 ± 10.3	—
FEF_25–75%_ (l s^−1^)	<0.001	3.59 ± 1.22	2.78 ± 1.06	0.015	2.46 ± 0.92	2.24 ± 0.81	ns	2.97 ± 1.22	2.49 ± 0.97	ns	3.03 ± 1.23	2.48 ± 0.97	0.005
PEF (l s^−1^)	<0.001	7.66 ± 1.54	7.03 ± 2.02	ns	5.93 ± 1.42	5.68 ± 1.58	ns	6.02 ± 1.76	6.51 ± 2.01	ns	7.28 ± 1.50	5.96 ± 1.69	0.046

Blood parameters													

WBC (*μ*l)	ns	6.77 ± 2.17	6.36 ± 1.67	—	5.97 ± 2.66	5.95 ± 1.62	—	5.23 ± 1.29	6.11 ± 1.79	—	7.12 ± 2.74	6.19 ± 1.41	—
RBC (millions mm^−3^)	<0.001	5.23 ± 0.35	4.99 ± 0.39	0.031	4.70 ± 0.59	4.60 ± 0.30	ns	5.00 ± 0.67	4.76 ± 0.39	ns	4.95 ± 0.47	4.82 ± 0.40	ns
Hemoglobin (gm dl^−1^)	<0.001	15.47 ± 0.95	14.68 ± 1.93	ns	13.54 ± 1.24	13.36 ± 1.12	ns	14.10 ± 1.70	13.78 ± 1.79	ns	14.72 ± 1.28	14.26 ± 1.44	ns
Hematocrit (%)	<0.001	49.0 ± 2.7	46.2 ± 6.2	ns	42.8 ± 3.8	41.7 ± 6.5	ns	44.2 ± 4.7	42.8 ± 7.8	ns	46.8 ± 4.2	45.3 ± 4.3	ns
Measures of oxidative stress													

8-OH-2dG (pg ml^−1^)	0.030	4335 ± 928	5845 ± 2559	0.022	8238 ± 5875	6300 ± 2554	0.040	7831 ± 6496	6035 ± 2631	ns	5347 ± 2577	6189 ± 2456	ns
8-iso-PGF2*α* (pg ml^−1^)	0.019	1371 ± 389	1809 ± 741	0.024	2387 ± 1201	2034 ± 1001	ns	2002 ± 1324	1849 ± 681	ns	1826 ± 808	2066 ± 1159	ns
o-Tyrosine (pg ml^−1^)	ns	4515 ± 6368	6248 ± 9770	—	13 753 ± 18 534	7687 ± 13 334	—	15 124 ± 20 269	7953 ± 13 669	—	5305 ± 7263	5560 ± 7941	—

Measures of pro-inflammation													

IL-6 (pg ml^−1^)	ns	0.998 ± 0.339	1.239 ± 1.434	—	1.668 ± 1.069	2.100 ± 5.811	—	1.498 ± 1.234	1.110 ± 0.635	—	1.263 ± 0.521	2.713 ± 7.119	—
IL-8 (pg ml^−1^)	ns	1.164 ± 0.351	1.716 ± 3.605	—	2.387 ± 2.939	15.646 ± 86.470	—	2.539 ± 3.360	2.697 ± 9.763	—	1.317 ± 0.514	20.582 ± 104.440	—
TNF*_α_* (pg ml^−1^)	ns	0.719 ± 1.188	1.254 ± 5.686	—	1.418 ± 2.579	3.741 ± 18.844	—	1.549 ± 2.891	0.925 ± 3.702	—	0.776 ± 1.180	5.441 ± 23.087	—
LTE4 (pg ml^−1^)	ns	3636 ± 1924	21 669 ± 85 719	—	36 968 ± 102 275	9558 ± 14 693	—	4062 ± 1407	18 277 ± 72 362	—	36 628 ± 102 404	9187 ± 11 363	—

Measures of anti-inflammation													

15-HETE (pg ml^−1^)	ns	2274 ± 950	2665 ± 1571	—	3600 ± 1334	2931 ± 1591	—	3115 ± 1527	2744 ± 1294	—	2895 ± 1218	2927 ± 1972	—
LXA4 (pg ml^−1^)	ns	3420 ± 841	4136 ± 1503	—	5433 ± 2192	4950 ± 2213	—	5021 ± 2266	4629 ± 1784	—	4081 ± 1657	4520 ± 2237	—
RvE1 (pg ml^−1^)	ns	3242 ± 1637	4188 ± 2316	—	6262 ± 4200	5235 ± 3653	—	5357 ± 4713	4378 ± 2526	—	4430 ± 2521	5373 ± 3910	—

Values: means ± SD.

Abbreviations: YOA, years of age; BMI, body mass index; FVC, forced vital capacity; FEV1, forced expiratory volume in 1 s; FEF25–75, forced expiratory flow from 25%–75% of FVC; PEF, peak expiratory flow; WBC, white blood cell concentration; RBC, red blood cell concentration; 8-OHdG, 8-hydroxy-2’-deoxyguanosine; 8-iso-PGF2*α*: 8-iso-prostaglandin F2*α*; IL-6: interleukin 6; IL-8: interleukin 8; TNFa: tumor necrosis factor alpha; LTE4: leukotriene E4; 15-HETE: 15 hydroxyeicosatetraenoic acid; 15-epi-LXA4:15-epi lipoxin A4; RvE1: resolvin E1.

Residual = absolute-predicted.

Further analysis using one-tailed *T* tests showed significant reductions in both height and weight in males with high arsenic exposure. Females exposed to high levels of arsenic were also shorter than females with lower exposure, although this was not statistically significant (*p* = 0.059). Males, females, and current smokers exposed to high arsenic levels had significant reductions in measured FVC and FEV_1_, while only females had significant decrements in percent predicted and residual FVC. Males with high arsenic exposure showed significant reductions in RBCs compared to males with low arsenic exposure. Across males and females, and never smokers and current smokers, high arsenic exposure resulted in non-significant decreases in RBC and hemoglobin concentrations, as well as hematocrit levels in all groups. Males exposed to high levels of arsenic had significant increases in oxidative stress measures 8-OH-2dG and 8-iso-PGF2*α*. In contrast, 8-OH-2dG levels were significantly reduced in females in the HighAE group.

### 3.2. Untargeted EBC analysis

**Figure 2.**
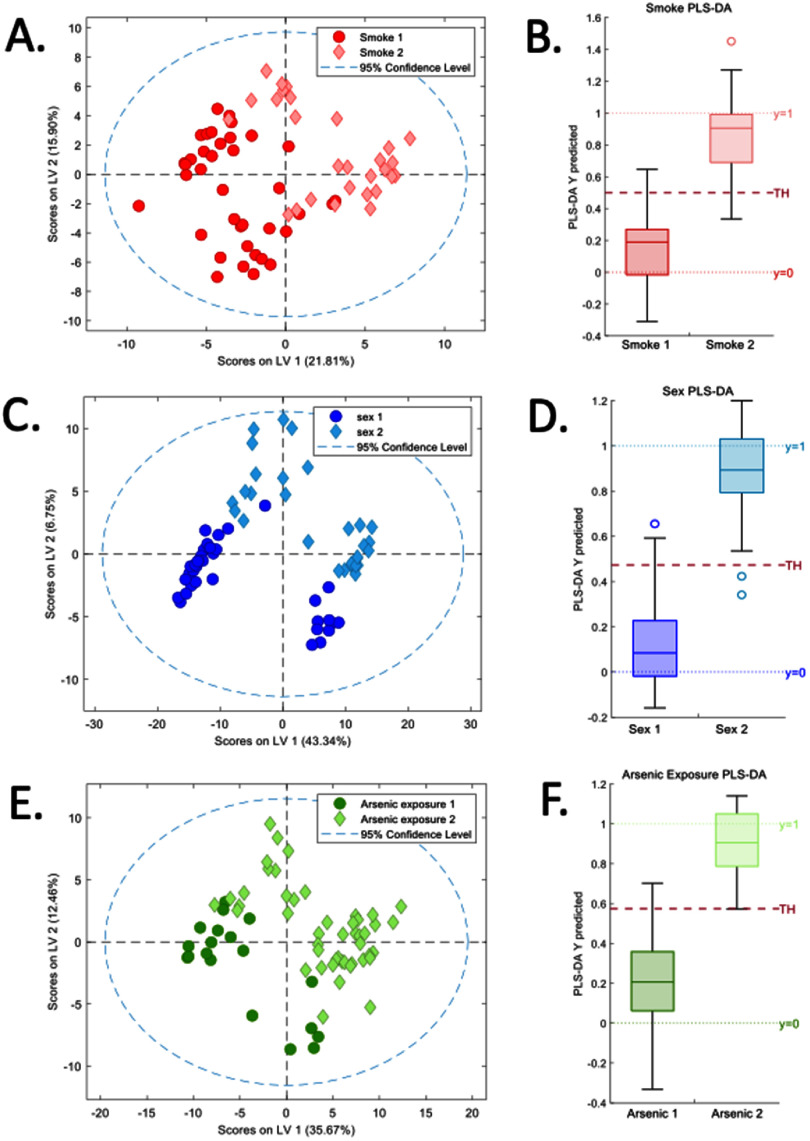
Score plots and box plots of PLS-DA models. The plots show the separation and distribution of the PLS-DA model output for the merged dataset for the grouping factors of smoking history (A) and (B), sex (C) and (D) and arsenic exposure (E) and (F).

PLS-DA generated three models for each dataset examined. These models identified peak intensities that were associated with stratified grouping characteristics of arsenic exposure, smoking history, and sex. The peak intensities, AUC, sensitivity, and specificity are shown in table 5. In most cases, some of the peaks associated with the grouping characteristics overlapped, resulting in 7 classes: sex, smoking, arsenic, sex + smoking, sex + arsenic, smoking + arsenic and all classes (sex + smoking + arsenic) (table 6). Of the 140 peaks in the EBC-HILIC data, PLS-DA identified 47 peak intensities associated with arsenic exposure, 56 peak intensities associated with smoking history, and 66 peak intensities associated with sex. Of the 140 peaks in the EBC-RP data, PLS-DA identified 78 peak intensities associated with arsenic exposure, 103 peak intensities associated with smoking history, and 154 peak intensities associated with sex. Of the 386 peaks in the EtOH-RP data, the PLS-DA identified 155 peak intensities associated with arsenic exposure, 144 peak intensities associated with smoking history, and 159 peak intensities associated with sex. Of the 210 peaks in the merged data, PLS-DA identified 65 peak intensities associated with arsenic exposure, 36 peak intensities associated with smoking history, and 113 peak intensities associated with sex. PLS-DA, using the merged data, produced the best discrimination with the highest AUC, sensitivity, and specificity (table 5). This also produced the lowest overlap between classes (table 6) for all grouping characteristics despite having the lowest number of samples available for analysis. This suggests that expanding the number and chemical and physical diversity of the compounds available for analysis outweighed the effect of the reduced sample number. The score and box plots in figure 2 show the separation and distribution of the PLS-DA model output for the merged dataset.

**Table 5.** Sample size, identified peak intensities, area under the curve (AUC), sensitivity, and specificity for all partial least squares discriminant analysis (PLS-DA).

Class	Data	Samples	Peaks	AUC	Sensitivity	Specificity
Smoke	Merged	66	36	0.93 (±0.04)	0.86 (±0.08)	0.83 (±0.10)
	EBC-HILIC	73	56	0.86	0.76	0.8
	EBC-RP	107	103	0.9	0.83	0.78
	EtOH-RP	106	144	0.9	0.81	0.79

Arsenic	Merged	66	65	0.97 (±0.03)	0.89 (±0.16)	0.88 (±0.06)
	EBC-HILIC	73	47	0.82	0.75	0.72
	EBC-RP	107	78	0.75	0.69	0.65
	EtOH-RP	106	155	0.84	0.71	0.78

Sex	Merged	66	113	0.95 (±0.04)	0.85 (±0.11)	0.90 (±0.09)
	EBC-HILIC	73	66	0.88	0.74	0.83
	EBC-RP	107	154	0.88	0.8	0.75
	EtOH-RP	106	159	0.87	0.78	0.78

**Table 6.** PLS-DA grouping characteristics (overlapping seven classes): sex, smoking, arsenic, sex + smoking, sex + arsenic, smoking + arsenic and all classes (sex + smoking + arsenic).

Data	Samples	Arsenic	Smoke	Sex	Arsenic + smoke	Arsenic + sex	Smoke + sex	Arsenic + smoke + sex
Merged	66	63	34	109	0	2	2	0
EBC-HILIC	73	29	39	47	6	8	7	4
EBC-RP	107	48	63	108	9	15	25	6
EtOH-RP	106	110	91	118	26	14	22	5

**Figure 3.**
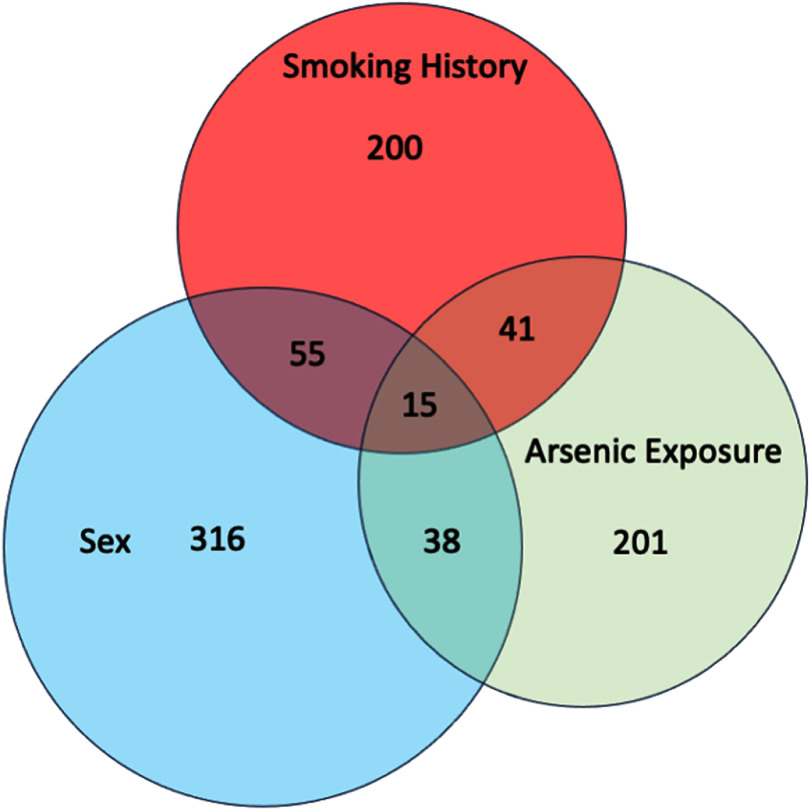
Venn diagram of peaks identified using merged results of PLS-DA. Diagram shows the EBC-HILIC data, EBC-RP data, EtOH-RP data and the merged data. 201 peaks were identified associated with arsenic exposure without any overlap with any other classes.

**Figure 4.**
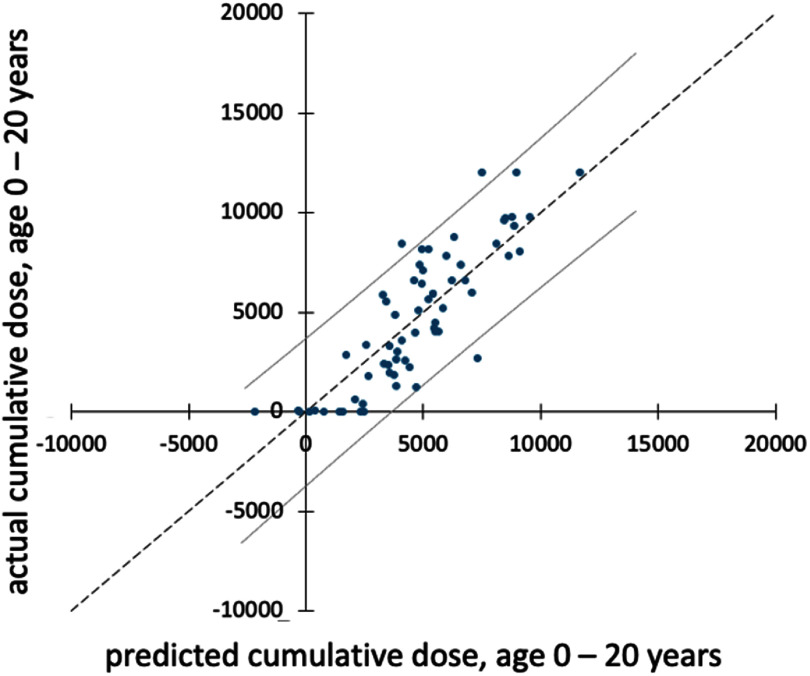
Plot of cumulative dose of arsenic versus the predicted cumulative dose of arsenic generated using step-wise linear regression analysis. Predicted cumulative dose of arsenic was significantly associated (*p* < 0.001) with the continuous variable of cumulative arsenic exposure from age 0–20. The best fit model contained 6 peaks with an adjusted *R* square of 0.81. This regression model contained 2 peaks from the EBC-HILIC data, 2 peaks from the EBC-RP data, and 2 peaks from the EtOH-RP data.

After merging the peaks from the four PLS-DA, 201 peaks associated with arsenic exposure without any overlap with any other class were identified (figure 3). Of the 201 total arsenic peaks, 29 peaks were from the EBC-HILIC data, 48 peaks were from the EBC-RP data, and 110 peaks were from the EtOH-RP data (table 6), while 15 non-overlapping peaks were from the merged data. Using these 201 peaks, step-wise linear regression analysis generated a model (figure 4) that was significantly associated (*p* < 0.001) with the continuous variable of cumulative arsenic exposure from age 0–20. The best-fit model contained six peaks, with an adjusted *R* square of 0.81. This regression model contains two peaks from the EBC-HILIC data, two peaks from the EBC-RP data, and two peaks from the EtOH-RP data (table 7). Based on the Type III sum of squares, the identified explanatory variable with a molecular weight of 350.2316 g and a retention time of 1.13 s was the most influential (table 7). Of these six peaks, the peak with a molecular weight of 138.0679 g and retention time of 2.90 s was identified as 2,6-Dimethyl-1,4-benzenediol with a confidence of 80.93%; the peak with a molecular weight of 479.305 g and retention time of 13.25 s was identified as glycerolphospholipid, LysoPE(0:0/18:1(9Z)), or LysoPE(0:0/18:1(11Z)) with a confidence of 80.25%. The remaining four identified peaks could not be identified with a confidence level greater than 80%.

**Table 7.** Step-wise linear regression analysis generated a model (figure 4) that was significantly associated (*p* < 0.001) with the continuous variable of cumulative arsenic exposure from age 0–20. The best fit model contained 6 peaks with an adjusted *R* square of 0.81.

Mol Wt	Retention time	Sample	Method	Compound	Molecular formula	Score	DOC	Chemical class	Beta	*t*	Pr > |*t*|	Lower bound (95%)	Upper bound (95%)
138.0437	2.34	EBC	HILIC	No ID	No MF	—	Up	Hydroquinones	0.196	3.253	0.002	0.075	0.316
138.0697	2.9	EBC	RP	2,6-Dimethyl-1,4-benzenediol	C8 H10 O2	80.93	Up	—	0.33	5.202	< 0.0001	0.203	0.456
274.2558	10.58	EtOH	RP	No ID	No MF	—	Up	—	0.153	2.362	0.021	0.023	0.283
350.2316*	1.13	EBC	HILIC	No ID	No MF	—	Up	—	0.487	7.512	< 0.0001	0.357	0.617
473.3074	11.54	EBC	RP	No ID	No MF	—	Down	—	−0.159	−2.799	0.007	−0.273	−0.045
479.305	13.25	EtOH	RP	LysoPE(0:0/18:1(9Z))/LysoPE(0:0/18:1(11Z))	C23 H46 N O7 P	80.25	Up	Glycerophospholipids	0.184	3.104	0.003	0.065	0.302

Using the stepwise variable selection method, six variables were identified with an *R* squared of 0.829. This indicates that 83% of the variability in the dependent variable CUM_AGE0_20 is explained by the six identified variables. Based on the Type III sum of squares, the identified explanatory variable with a molecular weight of 350.2316 Mz and retention time of 1.13 s is the most influential (*).

## 4. Discussion

In this study, we examined specific targeted metabolomic markers as well as untargeted metabolites in EBC, a measure of airway lining fluid composition, from individuals that were exposed to low or high levels of arsenic early in their lifetime. Our targeted markers included mediators of oxidative stress, inflammation, and lung remodeling. We found that HighAE affected height in males, pulmonary function measures in both males and females, and RBC concentrations in males. Two markers of oxidative stress, 8-OH-2dG and 8-iso-PGF2*α*, were significantly higher in males and 8-OH-2dG was significantly lower in females with high arsenic exposure. Untargeted analysis using LCMS on EBC revealed metabolomic markers that differentiated the HighAE group from the LowAE group, with a subgroup of markers whose concentrations were proportional to the level of arsenic exposure. These results show that early life exposure to arsenic has a lifelong effect that continues in the later years of life.

This was an initial study to examine the viability of using EBC analysis to identify persistent changes in the metabolic profile of individuals exposed to high arsenic levels in their drinking water early in their lives. We limited the number of subjects to 150. We recruited 75 males and 75 females born during the period when municipal water arsenic levels in Antofagasta were high (1958–1970). Subjects with low early life arsenic exposure were born and grew up in regions of Chile that did not experience elevated arsenic levels, but subjects from these two populations were not selectively recruited. In addition, we did not recruit participants based on their smoking histories. Not selectively recruiting subjects for arsenic exposure and smoking history resulted in an unequal number of subjects in subgroups defined by sex, arsenic exposure, and smoking history. There were fewer former smokers than never and current smokers, with only four LowAE females and five HighAE males. These small group sizes adversely affected the power of our analysis, making it impossible to reliably examine the effects of sex and arsenic exposure in former smokers. Therefore, we excluded former smokers from the final statistical analysis.

Subjects in this study that were exposed to high levels of arsenic early in life were significantly shorter and had significant decreases in measured, percent predictive and residual values for pulmonary function measures of FVC and FEV_1_ compared to the low arsenic exposure control group (table 2). Previous studies have shown an association between chronic arsenic exposure and reduced childhood height [36], and acute early life arsenic exposure and reduced adult height [37]. It is likely that the decreases in measured FVC and FEV_1_ can in part be explained by the observed arsenic associated decrease in height. However, when adjusted for height, the decreased percent predicted and residual FVC and FEV_1_, suggest that early life arsenic exposure adversely effects lung growth and development beyond an effect on overall growth. Percent predicted values for FVC and FEV_1_ were 6.07% and 7.36% lower, respectively, in the high arsenic group subjects. Despite significant *p* values obtained from the ANOVA and every subgroup (male, female, never smoker or current smoker) having lower mean values for percent predicted and residual FVC and FEV_1_, only the decrements in the female subgroup were significant for percent predicted and residual FVC. Dauphine *et al* [11], who examined the effects of high tap water arsenic exposure (>800 *µ*g l^−1^) in utero and up to the age of 10 years in individuals living in Antofagasta, Chile, observed a similar pattern in pulmonary function decrements for percent predicted and residual FVC and FEV_1_ where there was a significant overall effect, but only one subgroup had any significant effect. The pattern of pulmonary function decrements observed in this and Dauphine *et al*’s [11] study is likely due to the small number of subjects studied and the affect this has on the ability to analyze subgroup effects. This limitation should not, however, distract from the observation in both studies that early-life exposure to high arsenic levels in drinking has an overall effect of inducing pulmonary function decrements.

We also observed significant decrements in RBCs in males exposed to high arsenic levels and trends consisting of decreases in RBCs, hematocrit, and hemoglobin concentrations in both males and females in the HighAE groups. A correlational study by Heck *et al* [38] suggested that chronic arsenic exposure, as measured by urinary arsenic levels, is negatively correlated with hemoglobin concentrations in men and women. Our results were similar to those of Parvez *et al* [39], who showed that increased arsenic levels in drinking water were significantly correlated with decreased RBC counts and hematocrit levels in males.

We chose the targeted metabolites in this study based on their ability to indicate oxidative stress, pro- and anti-inflammation, and remodeling in the lungs. We also selected metabolites that were detected in the EBC. Of the 13 targeted metabolomic markers, we were able to measure ten in our EBC analysis. Certain caveats must be considered when interpreting the metabolites identified using our targeted and untargeted approaches. First, EBC contain a diverse range of compounds that originate from individual metabolic and non-metabolic processes. Non-volatile metabolites found in EBC are believed to originate from microdroplets of airway lining fluid produced in the deep lung and are diluted by condensed water vapor that originates in the upper and lower conducting airways [40]. The electrical conductivity was used as a measure of the electrolyte concentration in the EBC. The electrolyte concentrations in the deep lung airway lining fluid were similar to those found in plasma [41]. In our study, the individual subject’s dilution factors ranged from 100 to 670. As a result, a non-volatile metabolite that has a low concentration in the airway lining fluid is difficult to measure and may be below the level of detection. It is also possible that markers found to be below the level of detection were not detected in EBC. Three of the 13 targeted metabolites in this study, 11-dehydro-TXB_2_, MMP-9, and TIMP-1, were below detection levels. While non-volatile compounds in EBC represent an airway sample, the tissue origin of the targeted and untargeted metabolites is not necessarily the lungs. Volatile compounds in EBC, on the other hand, originate not only from the deep lung, but also from the upper airway and digestive tract and condense out of the vapor phase at low temperatures or adhere to the glass of the condensation tube. As with non-volatile compounds, volatile compounds in EBC originate from non-metabolic and metabolic processes [40].

The results of our targeted analysis showed that two measures of oxidative stress, 8-OH-2dG and 8-iso-PGF2*α*, were significantly increased in males in the HighAE group. 8-hydroxy-2’-deoxyguanosine is a widely studied marker of oxidative stress and carcinogenesis, specifically marking oxidative damage to DNA [42, 43]. 8-iso-prostaglandin F2*α* is an isoprostane produced by the perioxidation of arachidonic acid in cell membranes [44], also indicating oxidative stress and often seen in a variety of diseases such as acute coronary syndrome [45], neurological diseases such as Alzheimer’s and Parkinson’s disease, diabetes mellitus and hypercholesterolemia [46, 47]. Both are often detected in the urine of individuals exposed to high levels of arsenic, but we were able to detect them in EBC, making this the first time that we are aware that these metabolites were analyzed in EBC in early life arsenic-exposed individuals. Chronic arsenic exposure leads to higher levels of 8-OH-2dG in urine samples of children [48, 49] and adults [49], and is linked to oxidative stress [49], lung carcinogenesis, and cancer [11, 50]. 8-iso-prostaglandin F2*α* is also observed in the urine of males undergoing oxidative stress from polycyclic aromatic hydrocarbons and arsenic exposure [51]. In rats, two months of oral administration of tetraarsenic tetrasulfide (As_4_S_4_) resulted in a significant increase in dimethylarsinic acid (DMA) in the livers of rats, and subsequent lipidomic analysis showed significantly elevated levels of cyclooxygenase metabolites, including 8-iso-PGF2*α* [52].

Finding significantly greater amounts of these oxidative stress markers in the EBC of our male HighAE group may indicate that arsenic exposure continues to affect the lungs many years after the original exposure, although our subjects may have had high levels of these metabolites due to disease processes not associated with arsenic exposure [51–53]. Inorganic arsenic is primarily absorbed through the GI tract and is metabolized in the liver. It is methylated in the liver and ultimately forms dimethylarsinic acid (DMA), which is then excreted. DMA enters the blood and is distributed in various tissues of the body [53]. Animal studies [54, 55] have shown that the lung is one of the primary tissues for DMA uptake, and that it induces DNA strand breaks in mice and rats when administered orally or directly to lung cells [56]. Arsenic-derived oxidant injury has also been shown to affect cytochrome P450 enzyme-mediated arachidonic acid (AA) metabolism [57, 58]. Anwar-Mohamed *et al* [58] showed that administering an acute dose of arsenite (AsIII) to mice resulted in increased gene and protein expression of some cytochrome p450 enzymes in the lung, which in turn led to the metabolism of AA and an increase in AA breakdown products. They surmised that arsenic-induced inflammation might have been responsible for this phenomenon. In a 2017 meta-analysis of 58 studies, Xu *et al* [59] examined oxidative damage induced by arsenic exposure in mice and rats and found significantly increased levels of oxidant markers and reduced antioxidative substances, resulting in oxidative injury, including lipid peroxidation and DNA damage.

In our study, 8-OH-2dG levels were significantly lower in females in the HighAE group than in those in the LowAE group. This indicates that there was less oxidative DNA damage in females exposed to arsenic than in males, indicating that there may be sex-specific responses to arsenic exposure. Animal studies [60] have shown that in utero exposure to arsenic causes a sex-specific carcinogenic response, which may be due to changes in DNA methylation [61]. Human studies have also suggested that early life arsenic exposure may affect later-in-life disease [7, 10, 11] and that this may be sex-specific. Pilsner *et al* [62] examined the effect of prenatal arsenic exposure on global methylation in maternal and cord blood in mother/newborn pairs in Bangladesh. A methyl-incorporation assay of cord blood showed that male offspring had a positive correlation between arsenic exposure and DNA methylation, whereas female offspring had an inverse relationship between arsenic exposure and DNA methylation. As has been found to cause both hypo- and hyper-DNA methylation [63]. Winterbottom *et al* [64] examined the effect of prenatal exposure to arsenic on the fetal expression of genes encoding regulators of post-translational histone modifications, one of many epigenetic processes. Three-hundred and eleven pregnant mothers from the United States had their urine arsenic measured, and biopsy samples from the fetal portion of the placenta were collected at birth. Multiple candidate epigenetic regulator genes (*n* = 138) were examined. The placental genes significantly associated with the mother’s urine arsenic levels were different between female and male fetal placentas. Female placental genes (*n* = 3) that were significantly associated with arsenic exposure were not components of epigenetic regulators and did not overlap with significant male genes. Fifteen out of the 40 male placental genes were significantly associated with arsenic exposure and were components of epigenetic regulators.

Untargeted analysis of EBC was performed using both HILIC and RP, whereas the EtOH wash of the glass EBC collection tube was analyzed with RP alone. HILIC was used to capture hydrophilic molecules with high polarity, whereas RP captured low-polarity molecules that were present in the aqueous EBC or more hydrophobic molecules that adhered to the glass EBC collection tube. These hydrophobic molecules would include volatile molecules that condense directly on the dry ice-chilled surface of the collection tube early in the collection, or molecules that were initially contained in expired microdroplets that adhered to the glass EBC collection tube when the EBC was thawed prior to sampling. The molecules that adhere to the glass collection tube are large hydrophobic molecules that form hydrogen bonds and/or electrostatic interactions with inorganic glass surfaces [65]. As a result, over 5000 molecules identified using the three sampling/analysis combinations (EBC/HILIC, EBC/RP, and EtOH/RP) have a broad range of physical and chemical characteristics.

PLS-DA was used to determine whether the complex mixture of compounds contained specific metabolic signatures related to the stratified group characteristics of arsenic exposure (LowAE vs HighAE), smoking history (never smokers vs current smokers), and sex (male vs female). PLS-DA was applied to each of the three sample/method and merged datasets. PLS-DA indicated that there were grouping characteristic-specific metabolic profiles present in all four datasets. PLS-DA of the individual sample/analysis datasets indicated considerable overlap of the compounds contained in the metabolic profiles of each grouping characteristic. This overlap is consistent with the interaction between arsenic exposure and sex observed for the targeted oxidative stress markers 8-OH-2dG and 8-iso-PGF2*α*.

The merged dataset provided the best discrimination, with minimal overlap of the group profiles (tables 5 and 6). Critical to this study was the identification of a metabolic profile that is specific to early life arsenic exposure, suggesting that arsenic exposure in drinking water during development has long-term effects on metabolism more than 45 years after high arsenic exposure had ended. This can be seen in the targeted data discussed above, with significant decrements in pulmonary function in both males and females, decrements in RBCs in males, and increases in two measures of oxidative stress in males, one of which was significantly reduced in females with high arsenic exposure.

Stepwise linear regression identified six molecules with a significant association with cumulative arsenic exposure from ages 0–20 years (figure 4). These six molecules came from all three sample/analysis datasets, indicating their diverse chemical compositions (table 7). Two of the molecules were identified with greater than 80% confidence: 2,6-Dimethyl-1,4-benzenediol and LysoPE(0:0/18:1(9Z))/ LysoPE(0:0/18:1(11Z)). Each of these molecules increased in the EBC as cumulative arsenic exposure increased.

2,6-Dimethyl-1,4-benzenediol belongs to a class of organic compounds known as hydroquinones. It has been detected, but not quantified, in a few different foods such as broccoli, common pea, and pulses. It is unclear why the level of 2,6-Dimethyl-1,4-benzenediol in EBC is negatively correlated with increasing early life arsenic exposure. LysoPE(0:0/18:1(9Z))/ LysoPE(0:0/18:1(11Z)) is a lysophosphatidylethanolamine (LPE). Lysophosphatidylethanolamine levels have been shown to be lower in rats with acute lipopolysaccharide-induced lung injury [66] and sepsis [67]. LPEs are found in all tissues of the body, including extracellular fluid and cell membranes. Lysophosphatidylethanolamine is usually generated within cell membranes as a result of phospholipase *A*-type enzymatic activity on regular phospholipids, such as phosphatidylcholine and phosphatidic acid, although they can also be generated by the acylation of glycerophospholipids or phosphorylation of monoacylglycerols [68]. Extracellular LPEs are transported into cells through phospholipid ATPase. Once in the cytosol, they are incorporated into the ER or mitochondrial membrane through an Ale1 transport membrane, where phosphatidylcholine is generated [68]. LysoPE(0:0/18:1(9Z))/ LysoPE(0:0/18:1(11Z)), and LPEs in general, are associated with cellular membrane metabolism; however, their precise role in mammalian cell signaling and cell function processes related to early life arsenic exposure remains to be determined.

Four other molecules were also found to be significantly correlated with increasing cumulative doses of early life arsenic exposure. None of these four molecules were identified with a confidence greater than 80%. The concentrations of three of these molecules were positively correlated, and one was negatively correlated with early life arsenic exposure.

## 5. Conclusions

We identified indicators of the early effects of high arsenic exposure during childhood, including reduced height and pulmonary function, in a population of otherwise healthy non-smoking and smoking Chilean adults. Targeted and untargeted analyses of EBC using LC–MS indicated that adults exposed to high arsenic levels in drinking water in utero and during early childhood retained a modified metabolic profile 47 years after the end of exposure. The targeted EBC analysis showed sex differences in the presence of oxidative stress indicators in male, but not in female, subjects. These data illustrate the utility of EBC to identify altered metabolic profiles in response to exposure to environmental toxins.

## Data Availability

The subjects in this study did not provide consent to release their medical and exposure history used in the published analysis, as a result the raw data set used in this publication is not publicly available.

## References

[1] Ravenscroft P (2007). Predicting the global distribution of natural arsenic contamination of groundwater. Symposium on arsenic: the geography of a global problem. https://www.geog.cam.ac.uk/research/projects/arsenic/symposium/S1.2_P_Ravenscroft.pdf.

[2] United States Environmental Protection Agency (2000). Arsenic occurrence in public drinking water supplies. https://nepis.epa.gov/Exe/ZyNET.exe/P1004W96.TXT?ZyActionD=ZyDocument%26Client=EPA%26Index=2000+Thru+2005%26Docs=%26Query=%26Time=%26EndTime=%26SearchMethod=1%26TocRestrict=n%26Toc=%26TocEntry=%26QField=%26QFieldYear=%26QFieldMonth=%26QFieldDay=%26IntQFieldOp=0%26ExtQFieldOp=0%26XmlQuery=%26File=D%253A%255Czyfiles%255CIndex%20Data%255C00thru05%255CTxt%255C00000021%255CP1004W96.txt%26User=ANONYMOUS%26Password=anonymous%26SortMethod=h%257C-%26MaximumDocuments=1%26FuzzyDegree=0%26ImageQuality=r75g8/r75g8/x150y150g16/i425%26Display=hpfr%26DefSeekPage=x%26SearchBack=ZyActionL%26Back=ZyActionS%26BackDesc=Results%20page%26MaximumPages=1%26ZyEntry=1%26SeekPage=x%26ZyPURL.

[3] Steinmaus C M, Yuan Y, Smith A H (2005). The temporal stability of arsenic concentrations in well water in western Nevada. Environ. Res..

[4] Welch A H, Helsel D R, Focazio M J, Watkins S A, Chappell W R, Abernathy C O, Calderon R L (1998). Arsenic in ground water supplies of the United States.

[5] Ferreccio C, González C, Milosavjlevic V, Marshall G, Sancha A M, Smith A H (2000). Lung cancer and arsenic concentrations in drinking water in Chile. Epidemiology.

[6] Steinmaus C (2014). Increased lung and bladder cancer incidence in adults after in utero and early-life arsenic exposure. Cancer Epidemiol. Biomark. Prev..

[7] Steinmaus C M (2013). Drinking water arsenic in northern Chile: high cancer risks 40 years after exposure cessation. Cancer Epidemiol. Biomark. Prev..

[8] Smith A H, Marshall G, Roh T, Ferreccio C, Liaw J, Steinmaus C (2018). Lung, bladder, and kidney cancer mortality 40 years after arsenic exposure reduction. J. Natl Cancer I.

[9] Smith A H, Marshall G, Yuan Y, Ferreccio C, Liaw J, von Ehrenstein O, Steinmaus C, Bates M N, Selvin S (2006). Increased mortality from lung cancer and bronchiectasis in young adults after exposure to arsenic in utero and in early childhood. Environ. Health Perspect..

[10] Dauphiné D C, Ferreccio C, Guntur S, Yuan Y, Hammond S K, Balmes J, Smith A H, Steinmaus C (2011). Lung function in adults following in utero and childhood exposure to arsenic in drinking water: preliminary findings. Int. Arch. Occ. Environ. Health.

[11] Roh T, Steinmaus C, Marshall G, Ferreccio C, Liaw J, Smith A H (2018). Age at exposure to arsenic in water and mortality 30–40 years after exposure cessation. Am. J. Epidemiol..

[12] Shi H, Shi X, Liu K J (2004). Oxidative mechanism of arsenic toxicity and carcinogenesis. Mol. Cell Biochem..

[13] Lantz R C, Hays A M (2006). Role of oxidative stress in arsenic-induced toxicity. Drug Metab. Rev..

[14] Dutta K, Prasad P, Sinha D (2015). Chronic low level arsenic exposure evokes inflammatory responses and DNA damage. Int. J. Hygiene Environ. Health.

[15] Hu Y, Li J, Lou B, Wu R, Wang G, Lu C, Wang H, Pi J, Xu Y (2020). The role of reactive oxygen species in arsenic toxicity. Biomolecules.

[16] Ahmed S, Khoda S M-E, Rekha R S, Gardner R M, Ameer S S, Moore S, Ekström E-C, Vahter M, Raqib R (2011). Arsenic-associated oxidative stress, inflammation, and immune disruption in human placenta and cord blood. Environ. Health Perspect..

[17] Josyula A B, Poplina G S, Kurzius-Spencera M, McClellen H E, Kopplina M J, Sturup S, Clark Lantz R, Burgess J L (2006). Environmental arsenic exposure and sputum metalloproteinase concentrations. Environ. Res..

[18] Olsen C E, Liguori A E, Zong Y, Lantz R C, Burgess J L, Boitano S (2008). Arsenic upregulates MMP-9 and inhibits wound repair in human airway epithelial cells. Am. J. Physiol..

[19] Burgess J L, Kurzius-Spencer M, O’Rourke M K, Littau S R, Roberge J, Meza-Montenegro M M, Gutiérrez-Millán L E, Harris R B (2013). Environmental arsenic exposure and serum matrix metalloproteinase-9. J. Expo Sci. Environ. Epidemiol..

[20] Olivas-Calderón E, Recio-Vega R, Gandolfi A J, Lantz R C, González-Cortes T, Gonzalez-De Alba C, Froines J R, Espinosa-Fematt J A (2015). Lung inflammation biomarkers and lung function in children chronically exposed to arsenic. Toxicol. Appl. Pharm..

[21] Syslová K, Kačer P, Kuzma M, Pankrácová A, Fenclová Z, Vlčková S, Lebedová J, Pelclová D (2010). LC-ESI-MS/MS method for oxidative stress multimarker screening in the exhaled breath condensate of asbestosis/silicosis patients. J. Breath Res..

[22] Weimann A, Belling D, Poulsen H E (2002). Quantification of 8-oxo-guanine and guanine as the nucleobase, nucleoside and deoxynucleoside forms in human urine by high-performance liquid chromatography-electrospray tandem mass spectrometry. Nucl. Acids Res..

[23] Alfaro M F, Walby W F, Adams W C, Schelegle E S (2007). Breath condensate levels of 8-isoprostane and leukotriene B4 after ozone inhalation are greater in sensitive versus nonsensitive subjects. Exp. Lung Res..

[24] Instituto Nacional de Estadísticas, Gobierno de Chile (2017). Estadísticas Vitales Anuario 2015. https://www.ine.cl/docs/default-source/publicaciones/2017/anuario-de-estadisticas-vitales-2015.pdf.

[25] WHO (World Health Organization) (2022). Guidelines for Drinking-Water Quality: Fourth Edition Incorporating the First and Second Addenda.

[26] Ruffino B, Campo G, Crutchik D, Reyes A, Zanetti M (2022). Drinking water supply in the region of Antofagasta (Chile): a challenge between past, present and future. Int. J. Environ. Res. Public Health.

[27] Romero L, Alonso H, Campano P, Fanfani L, Cidu R, Dadea C, Keegan T, Thornton I, Farago M (2003). Arsenic enrichment in waters and sediments of the Rio Loa (Second Region, Chile). Appl. Geochem..

[28] Blanc P D, Eisner M D, Balmes J R, Trupin L, Yelin E H, Katz P P (2005). Exposure to vapors, gas, dust, or fumes: assessment by a single survey item compared to a detailed exposure battery and a job exposure matrix. Am. J. Ind. Med..

[29] Cotes J E (1987). Medical Research Council questionnaire on respiratory symptoms (1986). Lancet.

[30] Graham B L (2019). Standardization of spirometry 2019 update. An official American thoracic society and European respiratory society technical statement. Am. J. Respir. Crit. Care Med..

[31] LaVange L (2017). Spirometry reference equations from the HCHS/SOL (Hispanic Community Health Study/Study of Latinos). Am. J. Respir. Crit. Care Med..

[32] Zamuruyev K O (2016). Human breath metabolomics using an optimized non-invasive exhaled breath condensate sampler. J. Breath Res..

[33] Kruve A, Rebane R, Kipper K, Oldekop M-L, Evard H, Herodes K, Ravio P, Leito I (2015). Tutorial review on validation of liquid chromatography-mass spectrometry methods: part II. Anal. Chim. Acta.

[34] Aksenov A A, Zamuruyev K O, Pasamontes A, Brown J F, Schivo M, Foutouhi S, Weimer B C, Kenyon N J, Davis C E (2017). Analytical methodologies for broad metabolite coverage of exhaled breath condensate. J. Chromatogr. B.

[35] Fothergill D M, Borras E, McCartney M M, Schelegle E S, Davis C E (2023). Exhaled breath condensate profiles of U.S. Navy divers following prolonged hyperbaric oxygen (HBO) and nitrogen-oxygen (Nitrox) chamber exposures. J. Breath Res..

[36] Gardner R M, Kippler M, Tofail F, Bottai M, Hamadani J, Grandér M, Nermell B, Palm B, Rasmussen K M, Vahter M (2013). Environmental exposure to metals and children’s growth to age 5 years: a prospective cohort study. Am. J. Epidemiol..

[37] Yorifuji T, Matsuoka K, Grandjean P (2017). Height and blood chemistry in adults with a history of developmental arsenic poisoning from contaminated milk powder. Environ. Res..

[38] Heck J E, Chen Y, Grann V R, Slavkovich V, Parvez F, Ahsan H (2008). Arsenic exposure and anemia in Bangladesh: a population-based study. J. Occup Environ. Med..

[39] Parvez F (2017). Arsenic exposures alter clinical indicators of anemia in a male population of smokers and non-smokers in Bangladesh. Toxicol. Appl. Pharm..

[40] Hunt J (2007). Exhaled breath condensate: an overview. Immunol. Allergy Clin. North Am..

[41] Nielson D W (1986). Electrolyte composition of pulmonary alveolar subphase in anesthetized rabbits. J. Appl. Physiol..

[42] Graille M, Wild P, Sauvain J-J, Hemmendinger M, Guseva Canu I, Hopf N B (2020). Urinary 8-OHdG as a biomarker for oxidative stress: a systematic literature review and meta-analysis. Int. J. Mol. Sci..

[43] Kasai H (1997). Analysis of a form of oxidative DNA damage, 8-hydroxy-2’-deoxyguanosine, as a marker of cellular oxidative stress during carcinogenesis. Mutat Res..

[44] Kim J-Y, Lee J-W, Youn Y-J, Ahn M-S, Ahn S-G, Yoo B-S, Lee S-H, Yoon J, Choe K-H (2012). Urinary levels of 8-iso-prostaglandin F2α and 8-hydroxydeoxyguanine as markers of oxidative stress in patients with coronary artery disease. Korean Circ. J..

[45] Szułdrzyński K, Zalewski J, Machnik A, Żmudka K (2010). Elevated levels of 8-iso-prostaglandin F_2α_ in acute coronary syndromes are associated with systemic and local platelet activation. Pol Arch. Med. Wewn.

[46] Davı G (1999). In vivo formation of 8-Iso-Prostaglandin F_2α_ and platelet activation in diabetes mellitus. Effects of improved metabolic control and vitamin E supplementation. Circulation.

[47] Reilly M P, Pratico` D, Delanty N, DiMinno G, Tremoli E, Rader D, Kapoor S, Rokach J, Lawson J, FitzGerald G A (1998). Increased formation of distinct F_2_ isoprostanes in hypercholesterolemia. Circulation.

[48] Calatayud M, Farias S S, de Paredes G S, Olivera M, Carreras N Á, Giménez M C, Devesa V, Vélez D (2019). Arsenic exposure of child populations in Northern Argentina. Sci. Total Environ..

[49] Xu Y, Wang Y, Zheng Q, Li X, Li B, Jin Y, Sun X, Sun G (2008). Association of oxidative stress with arsenic methylation in chronic arsenic-exposed children and adults. Toxicol. Appl. Pharm..

[50] Putila J J, Guo N L (2011). Association of arsenic exposure with lung cancer incidence rates in the United States. PLoS One.

[51] Wang T, Feng W, Kuang D, Deng Q, Zhang W, Wang S, He M, Zhang X, Wu T, Guo H (2015). The effects of heavy metals and their interactions with polycyclic aromatic hydrocarbons on the oxidative stress among coke-oven workers. Environ. Res..

[52] Zhou J (2019). Lipidomic profiling of subchronic As _4_S_4_ exposure identifies inflammatory mediators as sensitive biomarkers in rats. Metallomics.

[53] Ren C, Zhou Y, Liu W, Wang Q (2021). Paradoxical effects of arsenic in the lungs. Environ. Health Prev. Med..

[54] Li J, Duan X, Dong D, Zhang Y, Zhao L, Li W, Chen J, Sun G, Li B (2017). Tissue-specific distributions of inorganic arsenic and its methylated metabolites, especially in cerebral cortex, cerebellum and hippocampus of mice after a single oral administration of arsenite. J. Trace Elem. Med. Bio.

[55] Kenyon E M, Del Razo L M, Hughes M F (2005). Tissue distribution and urinary excretion of inorganic arsenic and its methylated metabolites in mice following acute oral administration of arsenate. Toxicol. Sci..

[56] Yamanaka K, Okada S (1994). Induction of lung-specific DNA damage by metabolically methylated arsenics via the production of free radicals. Environ. Health Perspect..

[57] Anwar-Mohamed A, El-Sherbeni A A, Kim S H, Althurwi H N, Zordoky B N M, El-Kadi A O S (2012). Acute arsenic toxicity alters cytochrome P450 and soluble epoxide hydrolase and their associated arachidonic acid metabolism in C57Bl/6 mouse heart. Xenobiotica.

[58] Anwar-Mohamed A, El-Sherbeni A, Kim S H, Elshenawy O H, Althurwi H N, Zordoky B N M, El-Kadi A O S (2013). Acute arsenic treatment alters cytochrome P450 expression and arachidonic acid metabolism in lung, liver and kidney of C57Bl/6 mice. Xenobiotica.

[59] Xu M, Rui D, Yan Y, Xu S, Niu Q, Feng G, Wang Y, Li S, Jing M (2017). Oxidative damage induced by arsenic in mice or rats: a systematic review and meta-analysis. Biol. Trace Elem. Res..

[60] Waalkes M P, Ward J M, Liu J, Diwan B A (2003). Transplacental carcinogenicity of inorganic arsenic in the drinking water: induction of hepatic, ovarian, pulmonary, and adrenal tumors in mice. Toxicol. Appl. Pharm..

[61] Xie Y, Liu J, Benbrahim-Tallaa L, Ward J M, Logsdon D, Diwan B A, Waalkes M P (2007). Aberrant DNA methylation and gene expression in livers of newborn mice transplacentally exposed to a hepatocarcinogenic dose of inorganic arsenic. Toxicology.

[62] Pilsner J R (2012). Influence of prenatal arsenic exposure and newborn sex on global methylation of cord blood DNA. PLoS One.

[63] Islam R, Zhao L, Wang Y, Lu-Yao G, Liu L-Z (2022). Epigenetic dysregulations in arsenic-induced carcinogenesis. Cancers.

[64] Winterbottom E F (2019). Prenatal arsenic exposure alters the placental expression of multiple epigenetic regulators in a sex-dependent manner. Environ. Health.

[65] McKenzie M E (2017). Adhesion of organic molecules on silica surfaces: a density functional theory study. J. Phys. Chem..

[66] Wang T, Lin S, Liu R, Li H, Liu Z, Zhang X, Xu H, Li Q, Bi K (2020). Metabolomic profile perturbations of serum, lung, bronchoalveolar lavage fluid, spleen and feces in LPS-induced acute lung injury rats based on HPLC-ESI-QTOF-MS. Anal. Bioanal. Chem..

[67] Peng J, Qiu C, Zhang J, Xiao X (2023). Serum metabolite profiling reveals metabolic characteristics of sepsis patients using LC/MS-based metabolic profiles: a cross-sectional study. BMC Med. Genomics.

[68] Wishart D (2022). HMDB 5.0: the human metabolome database for 2022. Nucl. Acids Res..

